# Evaluation of reference genes for real-time quantitative PCR analysis in southern corn rootworm, *Diabrotica undecimpunctata howardi* (Barber)

**DOI:** 10.1038/s41598-019-47020-y

**Published:** 2019-07-24

**Authors:** Saumik Basu, Adriano E. Pereira, Daniele H. Pinheiro, Haichuan Wang, Arnubio Valencia-Jiménez, Blair D. Siegfried, Joe Louis, Xuguo ‘Joe’ Zhou, Ana Maria Vélez

**Affiliations:** 1 Department of Entomology, University of Nebraska-Lincoln, Lincoln, NE 68583 USA; 2 Department of Biochemistry, University of Nebraska-Lincoln, Lincoln, NE 68583 USA; 3 Federal University of Lavras, Lavras, MG Brazil; 4 Department of Agronomy and Horticulture, University of Nebraska-Lincoln, Lincoln, NE 68583-0915 USA; 5 Departamento de Producción Agropecuaria, Universidad de Caldas, Manizales, Colombia; 6 Entomology and Nematology Department, University of Florida, Gainesville, FL 32611-0620 USA; 7 Department of Entomology, University of Kentucky, Lexington, KY 40546-0091 USA; 8 Present Address: Department of Entomology, Washington State University, Pullman, WA 99164 USA; 9 Present Address: Plant Genetics Research Unit, USDA/ARS, University of Missouri-Columbia, Columbia, MO 65211-7020 USA

**Keywords:** Biotechnology, Plant sciences, RNAi

## Abstract

Quantitative reverse transcription PCR (RT-qPCR) is one of the most efficient, reliable and widely used techniques to quantify gene expression. In this study, we evaluated the performance of six southern corn rootworm, *Diabrotica undecimpunctata howardi* (Barber), housekeeping genes (HKG), *β-actin (Actin)*, *β-tubulin (Tubulin)*, *elongation factor* 1 *alpha* (*EF*1*α*), *glyceraldehyde-3 phosphate dehydrogenase* (*GAPDH*), *40 S ribosomal protein S9* (*RpS9*) and *ubiquitin-conjugating protein* (*Ubi*), under different experimental conditions such as developmental stage, exposure of neonate and adults to dsRNA, exposure of adults to different temperatures, different 3^rd^ instar larva tissues, and neonate starvation. The HKGs were analyzed with four algorithms, including geNorm, NormFinder, BestKeeper, and delta-CT. Although the six HKGs showed a relatively stable expression pattern among different treatments, some variability was observed. Among the six genes, *EF*1*α* exhibited the lowest Ct values for all treatments while *Ubi* exhibited the highest. Among life stages and across treatments, *Ubi* exhibited the least stable expression pattern. *GAPDH*, *Actin*, and *EF1α* were among the most stable HKGs in the majority of the treatments. This research provides HKG for accurate normalization of RT-qPCR data in the southern corn rootworm. Furthermore, this information can contribute to future genomic and functional genomic research in *Diabrotica* species.

## Introduction

The southern corn rootworm (SCR), *Diabrotica undecimpunctata howardi* (Barber), is a polyphagous/omnivorous plant pest found in the United States east of the Rocky Mountains, southern Canada, and northern Mexico^1,2^. SCR larvae feed on a wide variety of plants especially grasses, but its host range also includes cucurbits, peanuts, soybeans, cotton seedlings, dry beans, and maize^1–4^. SCR neonates can cause significant damage to corn seedling by feeding on the roots and drilling into the stem, subsequently killing the bud and reducing the maize stand^1^ to as much as 75%^5^. Furthermore, SCR has been used as a model insect to evaluate toxicity to different pesticides, including *Bacillus thuringiensis* (Bt) Berliner^6–8^, synthetic insecticides^9^, and double-stranded RNA (dsRNA)^10–14^.

RT-qPCR has become an important research tool for the quantification of gene expression in functional genomics and more recently for the evaluation of RNAi-mediated gene knockdown^15^. This efficient PCR-based assay is increasingly being utilized to compare gene expression, for which normalization through housekeeping genes (HKGs) is required. It is well known that the sensitivity, specificity and accuracy of RT-qPCR depend on several factors, including primer efficiency, the number of replications, but most importantly the choice of appropriate reference genes^16,17^. HKGs are expected to be stably expressed in all cells of an organism regardless of the physiological conditions^18,19^. Several studies suggest that some HKGs are differentially expressed depending on the experimental condition and no single reference gene is stably expressed and appropriate for all biological tissues or experimental conditions^18–22^. Therefore, identification of the characteristics of different HKGs under different environmental conditions, life stages and in different tissues is essential for accurate quantification of gene expression by RT-qPCR^23^.

In the last few years, several studies validating reference genes for genomic and molecular research in insects have been performed in a variety of biotic and abiotic experimental conditions especially developmental stage, tissue, and temperature range^15^. Most of the insects used in the studies are within the orders Hemiptera (i.e. aphids and whiteflies), followed by Lepidoptera (i.e. fall armyworm, Monarch butterfly, and the silk moth), Diptera (*Drosophila* spp., fruit flies, and mosquitoes) and Coleoptera (Colorado potato beetle, western corn rootworm, and lady beetles)^15^. Several plant pests have been used in these validation studies, including the sweet potato whitefly, *Bemisia tabaci* Gennadius^18^, the lepidopterans beet armyworm, *Spodoptera exigua* Hübner, silk moth, *Bombyx mori* L.^24^, tobacco cutworm, *S*. *litura* Fabricius^25^, oriental armyworm, *Mythimna separate* Walker^26^, the cotton and soybean aphids, *Aphis gossypii* Glover^27^ and *A*. *glycines* Matsumura^28^, respectively, the Asian longhorned beetle, *Anoplophora glabripennis* Motschulsky^29^, the leaf beetles *Chrysomela populi* L. and *Phaedon cochleariae* Fabricius^30^, the desert locust, *Schistocerca gregaria* Forsskål^31^, the cotton leafhopper, *Amrasca biguttula biguttula* Ishida^32^, and the pink spotted ladybeetle, *Coleomegilla maculata* De Geer^33^. The most used HKGs in these studies include *β-actin (Actin*), *β-tubulin (Tubulin)*, *glyceraldehyde-3 phosphate dehydrogenase (GAPDH)*, *elongation factor* 1 *alpha* (*EF1α*), and *ribosomal proteins*^15,17^.

The present study evaluated the efficiency of HKGs for SCR RT-qPCR-based studies. The selected genes are among the six most commonly reported genes in the literature^15,17^: *Actin*^34,35^, *Tubulin*^34^, *EF1α*^34^, *GAPDH*^34,36^, *40 S ribosomal protein S9* (*RpS9*)^34^ and *ubiquitin-conjugating protein* (*Ubi*)^17^. These HKGs were tested across different larval tissues (head, midgut, and carcass [integument + fat body]) and developmental stages (egg, first, second and third instar larvae, pupa, adult male and female). Additionally, SCR neonates and adults were exposed to different stressors, including ingested *Snf7* dsRNA, starvation, and a range of temperatures (8 °C, 24 °C and 36 °C).

## Results

### Primer specificity and efficiency

Primer efficiency values ranged between 94.8% and 104.8% for all primers tested and correlation coefficients (R^2^) ranged around 0.99 (Table 1). RT-qPCR products generated with each set of primers were confirmed by the presence of a single peak in melting curve analyses (Supp. Fig. S1) and visualized in a 1% agarose gel (Supp. Fig. S2).

**Table 1:** Primer sequences and accession number of the six candidate reference genes in SCR.

Gene	Primer sequences (5’-3’)	NCBI Accession No.	Function	Amplicon (bp)	E (%)	R^2^
*β-actin (Actin)*	F: CCAGCTGCTTCCATACCCAA R: TGCCAGTTCCAGTTCCCTAG	KX981996	Cell mortality, structure, and Integrity^34,35^	129	94.8	0.996
*40 S ribosomal protein S9 (RpS9)*	F: GTCCATATGAGAAGGCCCGTT R: GGACCAATCGACGTAGGAGTG	KX981997	Component of the 40 S subunit of ribosome^34^	195	99.5	0.997
*β-tubulin (Tubulin)*	F: TCTTCATGCCCGGATTTGCT R: CACAAGCCGCCATCATGTTC	KX981998	Microtubule structures^34^	117	102.5	0.987
*Elongation factor 1α (EF1α)*	F: AAGCACTCCAGGAAGCAGTAC R: TGGGTTGTTCTTGGTGTCTCC	KX981999	Bring aminoacyl-transfer RNA to the ribosome^34^	110	99.8	0.999
*glyceraldehydes-3-phosphate* *dehydrogenase (GAPDH)*	F: CCTCTGGAAAATTGTGGCGTG R: GCCCAAACGAACAGTCAAGTC	KX982000	Carbohydrate metabolism^34,36^	179	97.8	0.996
*Ubiquitin conjugating enzyme E2 (Ubi)*	F: AGATGCTGTAGTCGCTAGGC R: TGATGACAACGACACCCTGG	KX982001	Cell lysis by ubiquitination^17^	178	104.8	0.984

F: forward; R: reverse; E: percentage of amplification efficiency; R^2^: correlation efficiency.

### Cycle threshold (Ct) values and expression studies of the six reference genes

**Figure 1:**
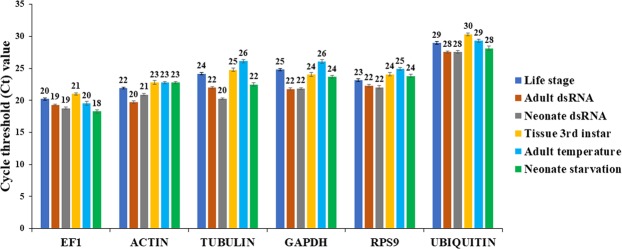
Cycle threshold (Ct) values generated with RT-qPCR for the HKGs in life stage, adult and neonate exposure to dsRNA, 3^rd^ instar larval tissues, adult exposure to different temperatures, and neonate starvation. HKGs evaluated: *Elongation factor 1α (EF1α)*, *β-Actin (Actin)*, *β-Tubulin (Tubulin)*, *40 S Ribosomal protein S9 (RpS9)*, *Glyceraldehydes-3-phosphate dehydrogenase (GAPDH)*, and *Ubiquitin conjugating protein (Ubi)*.

The Ct values generated in the RT-qPCR ranged between 18 and 30 throughout the treatments (Fig. 1). *EF1α* gene exhibited the lowest Ct values ranging from 18 to 20 for all treatments, and *Ubi* showed the highest Ct values in all treatments ranging from 28 to 30 (Fig. 1). *Actin* exhibited the second lowest Ct values (20–23), and Ct values for the genes *GAPDH*, *Tubulin*, and *RpS9* ranged from 20 to 26 (Fig. 1). To perform the HKG validation study, we verified and compared the expression levels of each HKG among different SCR developmental stages (Supp. Fig. S3). In general, all HKGs were expressed in each stage at different levels, with the highest expression levels observed in adults especially in males (except *RpS9*) followed by eggs (*GAPDH*, *Actin*, and *EF1α*) and pupa (*GAPDH*, *Ubi*, and *RpS9*) (Supp. Fig. S3). *RpS9* was the only HKG gene assayed that showed lower expression in males compared to females (Supp. Fig. S3).

### Stability of candidate reference genes

**Figure 2:**
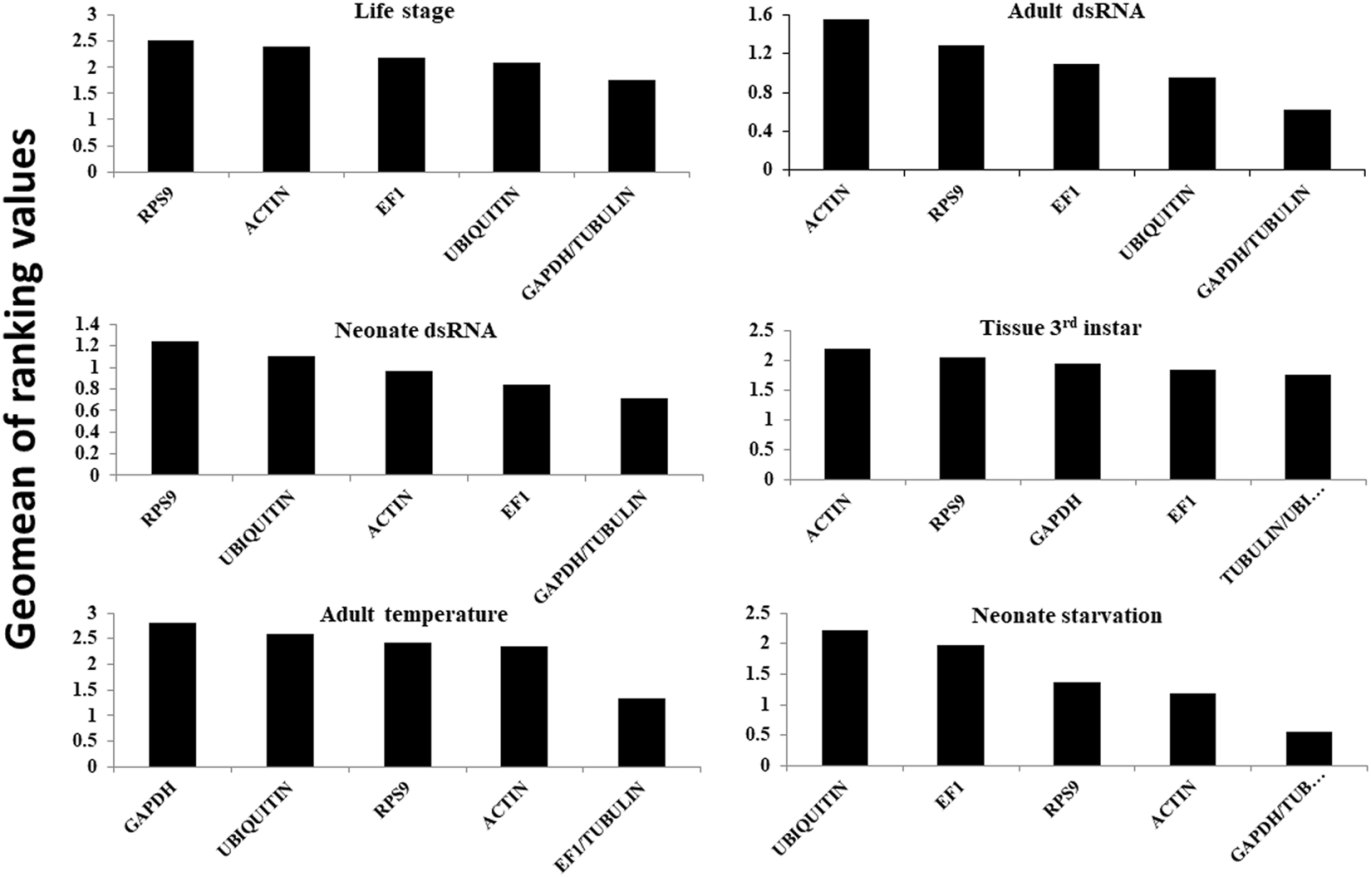
Stability of candidate reference genes according to geNorm in each bioassay: life stage, adult dsRNA exposure, neonate dsRNA exposure, 3^rd^ instar larval tissues, adult exposed to different temperatures, and neonate starvation. A lower Geomean value suggests stable expression.

Four different algorithms were used (geNorm, NormFinder, BestKeeper, and delta-CT) for the validation of stability of the six candidate HKGs evaluated under six different experimental conditions (i.e. developmental stages, adult exposure to dsRNA, neonate exposure to dsRNA, 3^rd^ instar tissue types, effect of temperature range to adults, and neonate starvation), using the web-based tool RefFinder which provides ranking for HKGs. The final ranking of the HKGs was calculated from the geometric mean of all the four different programs used in this study (Fig. 2). A lower geometric mean value represents higher stability of the HKGs.

**Figure 3:**
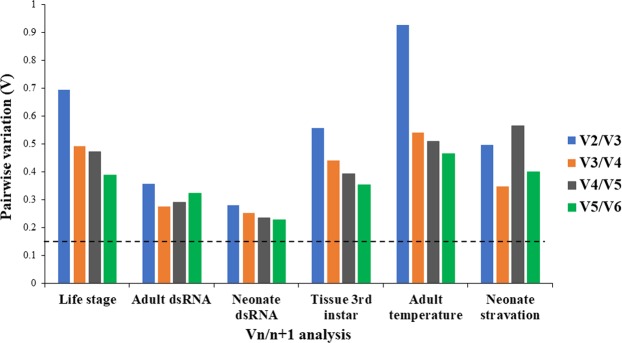
Optimal number of reference genes required for accurate normalization of gene expression for each bioassay. Based on geNorm analysis, average pairwise variations are calculated between the normalization factors NF_n_ and NF_n + 1_. Values < 0.15 indicate that additional genes are not required for the normalization of gene expression.

For the developmental stages, *GAPDH* and *Actin* were the most stable HKGs based on RefFinder ranking values, 1.32 and 1.68, respectively (Table 2). In the adults exposed to *Snf7* dsRNA, *EF1α* and *GAPDH* were the most stable HKGs based on RefFinder ranking values, 1.19 and 1.57, respectively (Table 3). Whereas in the neonates exposed to *Snf7* dsRNA, *GAPDH* and *Tubulin* were the most stable HKGs with RefFinder ranking values 1.57 and 2.21, respectively (Table 4). For the 3^rd^ instar larval tissues, *GAPDH* and *Actin* were the most stable HKGs with RefFinder ranking values 2.00 and 2.06, respectively (Table 5). For exposure of adults to different temperature (8, 24, or 36 °C), *RpS9* and *Actin* were the most stable HKGs with RefFinder ranking values 1.19 and 1.41, respectively (Table 6). For neonate starved for 48 h, *Actin* exhibited the lowest value followed by *GAPDH* with RefFinder ranking values 1.32 and 1.68, respectively (Table 7). One interesting observation obtained from this study was that the *Ubi* gene exhibited the highest stability values, but was the least stable HKG across all treatments in all algorithms (Tables 2–7**)** except for BestKeeper in adults exposed to dsRNA (Table 3). The lowest index rankings of *Ubi* across all the treatments were consistent with the highest delta-Ct values generated from RT-qPCR (Fig. 1). We also calculated the optimal HKG number based on geNorm algorithm analysis of reference genes necessary for accurate normalization for each bioassay. We found that the recommended number of HKGs for SCR is at least two for each treatment based on the pairwise values that were >0.15, performed in geNorm (Fig. 3).

**Table 2:** Ranking of candidate HKGs based on stability values performed by RefFinder, Delta Ct, BestKeeper, NormFinder, and geNorm in SCR life stage (egg, neonate, 2^nd^ instar, 3^rd^ instar, pupa, adult male and female).

HKG Gene	Stability (RefFinder)	Rank	Stability (delta Ct)	Rank	Stability (best keeper)	Rank	Stability (normFinder)	Rank	Stability (geNorm)	Rank
*GAPDH*	1.32	1	3.33	1	2.02	3	2.01	1	3.11	1
*β-Actin*	1.68	2	3.55	2	1.81	2	2.49	2	3.11	2
*EF1α*	2.45	3	3.60	4	1.78	1	2.57	3	3.16	3
*β-tubulin*	4.16	4	3.57	3	2.43	5	2.59	4	3.48	5
*RpS9*	4.47	5	3.65	5	2.25	4	2.67	5	3.20	4
*Ubi*	6.00	6	3.78	6	2.64	6	2.96	6	3.58	6

**Table 3:** Ranking of candidate HKGs based on stability values performed by RefFinder, Delta Ct, BestKeeper, NormFinder, and geNorm in SCR adults exposed to *Snf7* dsRNA.

HKG Gene	Stability (RefFinder)	Rank	Stability (delta Ct)	Rank	Stability (best keeper)	Rank	Stability (normFinder)	Rank	Stability (geNorm)	Rank
*EF1α*	1.19	1	1.67	1	1.17	1	0.43	2	0.87	1
*GAPDH*	1.57	2	1.68	2	1.46	3	0.34	1	0.87	2
*β-tubulin*	2.71	3	2.02	3	1.20	2	1.38	3	1.22	3
*β-Actin*	4.43	4	2.20	4	1.94	6	1.66	4	1.49	4
*RpS9*	4.73	5	2.24	5	1.58	4	1.68	5	1.69	5
*Ubi*	5.73	6	3.06	6	1.73	5	2.80	6	2.14	6

**Table 4:** Ranking of the candidate HKGs based on stability values performed by RefFinder, Delta Ct, BestKeeper, NormFinder, and geNorm in SCR neonates exposed to *Snf7* dsRNA.

HKG Gene	Stability (RefFinder)	Rank	Stability (delta Ct)	Rank	Stability (best keeper)	Rank	Stability (normFinder)	Rank	Stability (geNorm)	Rank
*GAPDH*	1.57	1	2.60	1	1.11	2	0.74	1	1.33	3
*β-tubulin*	2.21	2	2.95	3	1.04	1	1.24	2	2.05	4
*β-Actin*	2.21	3	2.91	2	1.49	4	1.95	3	1.26	1
*EF1α*	2.78	4	3.08	4	1.31	3	2.26	5	1.26	2
*RpS9*	4.73	5	3.32	5	2.27	5	2.18	4	2.38	5
*Ubi*	6.00	6	5.36	6	2.38	6	5.08	6	3.37	6

**Table 5:** Ranking of the candidate HKGs based on stability values performed by RefFinder, Delta Ct, BestKeeper, NormFinder, and geNorm in SCR 3^rd^ instar larval tissues.

HKG Genes	Stability (RefFinder)	Rank	Stability (delta Ct)	Rank	Stability (best keeper)	Rank	Stability (normFinder)	Rank	Stability (geNorm)	Rank
*GAPDH*	2.00	1	4.30	2	2.06	2	2.96	4	2.23	2
*β-Actin*	2.06	2	4.16	1	2.15	3	2.46	2	2.33	3
*EF1α*	2.11	3	4.58	4	1.42	1	3.48	5	2.23	1
*β-tubulin*	4.16	5	4.59	5	2.41	4	2.90	3	3.87	5
*RpS9*	2.78	4	4.39	3	2.69	5	2.41	1	3.53	4
*Ubi*	6.00	6	6.57	6	3.76	6	6.01	6	4.77	6

**Table 6:** Ranking of the candidate HKGs based on stability values performed by RefFinder, Delta Ct, BestKeeper, NormFinder, and geNorm in SCR adults kept at different temperatures for 8 h (8, 24, or 36 °C).

HKG Genes	Stability (RefFinder)	Rank	Stability (delta Ct)	Rank	Stability (best keeper)	Rank	Stability (normFinder)	Rank	Stability (geNorm)	Rank
*RpS9*	1.19	1	4.21	1	1.79	2	2.48	1	1.94	2
*β-Actin*	1.41	2	4.30	2	1.60	1	2.75	2	1.94	1
*EF1α*	3.22	3	4.54	3	2.62	4	2.94	3	2.87	3
*GAPDH*	3.72	4	4.72	4	2.31	3	3.14	4	3.41	4
*β-tubulin*	5.00	5	5.00	5	2.67	5	3.44	5	4.08	5
*Ubi*	6.00	6	6.45	6	3.79	6	5.80	6	4.87	6

**Table 7:** Ranking of the candidate HKGs based on stability values performed by RefFinder, Delta Ct, BestKeeper, NormFinder, and geNorm in SCR neonates starved for 48 h.

HKG Genes	Stability (RefFinder)	Rank	Stability (delta Ct)	Rank	Stability (best keeper)	Rank	Stability (normFinder)	Rank	Stability (geNorm)	Rank
*β-Actin*	1.32	1	2.53	1	1.00	1	0.78	1	1.60	3
*GAPDH*	1.68	2	2.90	2	1.35	2	1.62	2	1.56	1
*RpS9*	2.28	3	2.96	3	1.80	3	1.76	3	1.56	2
*β-tubulin*	4.47	4	3.77	4	2.13	5	2.97	4	2.89	5
*EF1α*	4.47	5	3.86	5	1.84	4	3.22	5	2.27	4
*Ubi*	6.00	6	4.46	6	2.51	6	3.94	6	3.41	6

## Discussion

Real Time-qPCR is one of the most valuable and reliable research tools used to quantify the expression of a target gene under different experimental conditions. Proper normalization of reference genes is necessary to get a robust and reliable estimate of gene expression under different experimental conditions and avoid unwanted variation. The HKGs evaluated in this study are reported to express constitutively to maintain various cellular functions. However, no HKG has been identified to be stably expressed in all tissues or cell types across different environmental conditions^15^. Therefore, the selection of appropriate HKGs under a particular treatment is important when using RT-qPCR to quantify gene expression. Many gene expression studies use a single endogenous control for different treatments and life stages, which can influence the statistical results and may lead to erroneous data interpretation^37^. Several genes have been selected as HKGs across different species, life stages, and tissues in various treatments^38^. Lack of stable expression of reference genes has been reported among different variables^16,39^ and suggests that no single gene (“universal reference gene”) can be selected for all variables^18,35,40,41^.

The results obtained in this study demonstrate that the stability of a HKG (among all six SCR HKGs studied) can be different under diverse experimental conditions including developmental stage, exposure of neonates and adults to dsRNA, exposure of adults to different temperatures, different 3^rd^ instar larva tissues, and neonate starvation (Tables 2–7; Figs 1–3**)**. The HKGs evaluated in this study have been used in a variety of organisms for evaluating the expression of target genes and can be found in several peer-reviewed publications reviewed in Lü *et al*.^15^. Among the six HKGs studied in this manuscript, we found that *Actin* and *GAPDH* were the most stable in different experimental conditions. *Actin* and *GAPDH* have been widely used as reference genes in different organisms ranging from insects to human tissues^15,42–44^. However, many studies have reported these genes to be unsuitable for RT-qPCR because of the variability of expression in different experimental settings and tissues^43–45^. In our study, *GAPDH* was found the most stable HKG in three of the total six experimental conditions (Tables 2,5,7) and exhibited the second highest Ct values (22–25) across four of the six treatments after *Ubi* (Fig. 1). *Actin* was the second most stable HKG in four treatments and the most stable in one of the six treatments (Tables 2, 5–7**)**. The role of *Actin* is to control cellular mobility and growth and helps in regulating G-protein pool^34,35^, while the *GAPDH* enzyme is a key Glycolysis/neo-glucogenic enzyme^34,36^. The results of this study suggest that the role of *Actin* and *GAPDH* seems to remain stable under different environmental conditions and tissues in SCR. Therefore, *Actin* and *GAPDH* can be used as reliable HKGs in RNAi-based experimental approaches and gene expression bioassays in SCR.

On the contrary, both *Ubi* and *RpS9* were the two least stable HKGs across all treatments and in five treatments, respectively (Tables 2–7**)** and are not recommended to be used for SCR gene normalization under the conditions mentioned above. In the 3^rd^ instar larval tissue, adult exposed to different temperatures and in neonates exposed to starvation, *Tubulin* was identified as the most stable HKG with the lowest calculated Geomean values using geNorm algorithm (Fig. 2). Although *Tubulin* has been reported to be used for gene expression studies in multiple organisms, its low stability in various life stages and different treatment conditions used in this study make it unsuitable as a “universal” HKG in SCR. The last HKG evaluated in this study, *EF1α*, was identified as the most stable gene in SCR adults exposed to *Snf7* dsRNA with lowest RefFinder ranking value (1.19) (Table 3).

Similar studies have been performed in other organisms, including insects, to evaluate the choice of reference gene selection for studying gene expression using RT-qPCR. Pan *et al*.^46^ found *GAPDH* as the HKG with the second highest Ct values across six different treatments performed in the lady beetle, *Hippodamia convergens* Guérin-Méneville, although was ranked as the most stable HKG in different treatments using different algorithms. Pan *et al*.^47^ also reported *GAPDH* as having the highest Ct values in the Monarch butterfly, *Danaus plexippus* L, but was ranked the most stable HKG in only one treatment (dsRNA). On the other hand, Yang *et al*.^48^ reported *Actin* as one of the least stable HKGs in the lady beetle *Harmonia axyridis* Pallas under biotic and abiotic conditions, while Dai *et al*.^44^ reported *Actin* as the most stable HKG under short-term thermal stress in the white fly *B*. *tabaci*. Rodrigues *et al*.^49^ validated the same HKGs used in this study (except *Ubi*) in western corn rootworm (WCR) and reported similar RT-qPCR Ct values, with *EF1α* exhibiting the lowest Ct values and *RpS9* exhibiting the highest Ct values in all four experiments (larval tissues, developmental stages, dsRNA exposure, and Bt protein exposure). However, Rodrigues *et al*.^49^ reported *EF1α* as the most stable among the five HKGs in four algorithms for two of the four experiments (3^rd^ instar larva tissue and life stage) and *Actin* as the least stable among five HKGs in one experiment (larval tissue) and the second least stable HKG in two (RNAi and Bt exposure) of the four experiments conducted. Even though SCR and WCR are similar and congeneric species, WCR is a pest that feeds almost exclusively on maize plants^50^, while SCR is polyphagous and is reported to feed on more than 200 different plant species from several families^1,2^. In addition, WCR is univoltine and eggs undergo diapause during the winter^2^, while SCR can have 3–4 generations per year in the field. These biological discrepancies can potentially play a role in the differences in HKG expressions reported in our study. Therefore, because of the differences in biology, feeding habits, oviposition, environmental factors and the differences in the methodology used between the two studies could lead to different results. Other studies performed with WCR have used similar HKGs to compare the expression of potential genes involved in Bt resistance (*EF1α*, *Actin*, and *GAPDH*)^51^, to compare candidate markers for behavioral resistance to crop rotation (*EF1α*)^52^, and to compare gene expression after gene knockdown by dsRNA (*Actin*)^53^. The examples provided above suggest that all of these HKGs can be selected for gene normalization, but only under specific condition(s) and should not be used as universal across all the different experimental conditions in any particular organisms.

Similar to the results of the studies listed above, our analyses also provide different results, for all four algorithms, under different experimental conditions, although the results were similar among some of the treatments. However, the most common observation from the analyses showed that *Ubi* was the least stable HKG across all evaluated treatments and in most algorithms (Tables 2–7**)**. Our results suggest that the recommended number of HKG is at least two for each treatment based on the pairwise values (>0.15) obtained with geNorm (Fig. 3). A single HKG is not recommended for SCR, similar to what has been suggested in most of the studies reported so far with other insects^45,54^. Based on our results and the results obtained in other organisms, it is evident that the expression of these HKGs varies based on experimental conditions. Therefore, there is no universal reference gene/internal control that can serve as the perfect gene in all experimental conditions which firmly implicates the necessity for the custom case-specific selection and evaluation of endogenous reference genes using RT-qPCR in different organisms including the SCR^15^.

In conclusion, we tested six different reference candidate genes in six different experimental conditions using four different statistical algorithms and the additional web-based computational platform, RefFinder, that integrates the four algorithms to offer the overall ranking of the stability of the six HKGs. The current study not only demonstrates a standardized procedure for studying SCR gene expression but also the selection of appropriate internal control for efficient gene normalization under different experimental conditions. *GAPDH* and *Actin* were the most stable HKGs under different experimental conditions and can be recommended for RNAi studies in SCR. *Actin* has been used as a standard HKG in most of the RNAi studies with corn rootworms, and has proved to generate reliable results^13,14,53,55–61^. *Ubi* and *RpS9* were considered as the least stable HKGs across the treatments which coincided with the higher Ct values generated in all treatments. Given that corn hybrids expressing *Snf7* dsRNA will soon be available to growers for the management of WCR^62^, and since SCR *Snf7* shares > 97% gene identity^13^ with WCR *Snf7*, it is likely that the hybrids will also affect SCR, and having a stable HKG will be necessary for studies in the upcoming years. This study can also be used as a steppingstone for providing detailed functional and genomic insights on SCR, an emerging model among belowground corn pests and several other economically important crops. Finally, two HKGs are recommended for normalization of SCR gene expression for each bioassay based on average pairwise variations (>0.15) calculated from geNorm analysis. Furthermore, the availability of HKGs and their stability will allow performing future studies focusing on genes essential for SCR biology, and choosing a reliable and appropriate HKGs will provide more accurate assessment of gene expressions for both the WCR and SCR.

## Materials and Methods

### Biological samples and experimental conditions

Newly emerged SCR adults were purchased from French Agricultural Research, Inc. (Lamberton, Minnesota) and kept in 30 cm^3^ BugDorm^®^ cages (MegaView Science Co., Ltd., Taichung, Taiwan) at 24–26 °C, 50–70% relative humidity (RH), and 14:10 (light:dark) photoperiod, provided with artificial diet and water until used for bioassays. Eggs were also purchased from French Agricultural Research, Inc. and kept in a dark incubator at 28 °C and 90% RH until hatching.

### Treatments

The treatments used to test the HKGs in this research were (1) developmental stage (egg, neonate, 2^nd^ instar, 3^rd^ instar, pre-pupa, pupa, and adult male and female), (2) adult exposure to *Snf7* dsRNA on artificial diet, (3) neonate exposure to *Snf7* dsRNA on artificial diet, (4) adult exposure to different temperatures (8 °C, 24 °C and 36 °C), (5) 3^rd^ instar larvae tissue (dissection of head, midgut, and carcass), and (6) neonate starvation for 48 h.

In all bioassays, three tubes (1.5 ml or 2 ml microcentrifuge tubes) containing the insects were collected per each treatment from each of the three different cohorts, having a total of 9 biological replicates. For developmental stages, approximately 100 eggs and 30 neonates were collected in 1.5 ml microcentrifuge tubes; 10–2^nd^ instar larvae, 5–3^rd^ instar larvae, 2-pupae, and 2-adults were collected in 2 ml microcentrifuge tubes, flash-frozen in liquid nitrogen, and placed at −80 °C until used for RNA extraction. For larval body tissues, 3^rd^ instar larvae were dissected to extract head, midgut, and carcass. Twenty heads, 5-midguts, and 5-carcasses were collected in 2.0 ml microcentrifuge tubes and flash-frozen in liquid nitrogen and subsequently stored at −80 °C until used for RNA extraction.

In dsRNA exposure bioassays, the gene used as target for dsRNA treatment was *Snf7*^13^, which is a key operator gene of the endosomal sorting complex (ESCRT) required for unconventional secretion in Eukaryotes^63^ and related to membrane molecule transport and membrane stability^64^. For adults, 12 artificial diet pellets (modified from Branson & Jackson^65^ and described in Khajuria *et al*.^57^) cut with a cork borer (4.0 mm diameter × 2.0 mm height; area = 0.1256 cm^2^) were placed in each of three 5.0 cm × 2.5 cm × 2.5 wells (one well per treatment) in a 16-well tray (C-D International, Pitman, NJ). The diet pellets in each well were treated with 3 µl of dsRNA solution to yield a concentration of 20 ng/cm^2^ of *Snf7* dsRNA or 20 ng/cm^2^ of Green Fluorescent Protein (*GFP*) dsRNA, or nuclease-free water only, and allowed to air dry. Ten SCR adults were placed in each of the three wells and allowed to feed for five days. Adults were transferred to clean trays with freshly treated diet on day 3. After five days, three 1.5 ml microcentrifuge tubes each containing 1–2 SCR adults were collected per well (treatment), flash-frozen in liquid nitrogen and stored at −80 °C until used for RNA extraction. For larvae, three 5.0 cm diameter petri dishes (PALL Corporation, Port Washington, NY, # 7242), each containing approximately 1 ml of SCR larval artificial diet (Frontier Agricultural Sciences^®^, Newark, DE) were treated with 450 µl of dsRNA solution that yielded 5.0 ng/cm^2^ of *Snf7* dsRNA or 5.0 ng/cm^2^ of *GFP* dsRNA, or nuclease-free water only, and allowed to air dry. One hundred SCR neonates (<24 h old) were transferred to each of the three petri dishes and allowed to feed for three days. After three days, three 1.5 ml microcentrifuge tubes each containing 20 neonates were collected per each petri dish (treatment), flash-frozen in liquid nitrogen and stored at −80 °C until used for RNA extraction. The *Snf7* dsRNA concentrations used for adults (20 ng/cm^2^) and neonates (5 ng/cm^2^) feeding assays were previously calculated and are within the concentration range that kills 50% (LC_50_) of the insects tested^13^.

To study the effect of starvation on gene expression of HKGs, SCR neonates (<24 h of hatching) were left starving in a 9.0 cm diameter × 1.4 cm height petri-dishes (Fisher Scientific, Pittsburgh, PA) lined with moist filter-paper for 48 h. Starved neonates were compared with newly hatched neonates. Three 1.5 ml microcentrifuge tubes containing 20–30 neonates each were collected in each treatment, flash-frozen in liquid nitrogen and stored at −80 °C until used for RNA extraction.

To elucidate the effect of heat and cold, SCR mixed adults (both males and females) were placed in 6.0 × 6.0 × 8.0 cm clear plastic boxes (Althor Products LLC, Windsor Locks, CT) and exposed to 8 °C, 24 °C, or 36 °C for 8 h before flash-frozen in liquid nitrogen and stored at −80 °C until used for RNA extraction.

### Housekeeping genes

The HKGs used in this research were the same evaluated by Rodrigues *et al*.^49^ in WCR: *Tubulin*, *EF1α*, *GAPDH*, *Actin*, and *RpS9* plus *Ubi* (Table 1). The primers for qPCR of the six reference genes were generated using Primer3^66^ and the primer efficiencies (E) and correlation coefficients (R^2^) were also calculated (Table 1**)**. For primer designing, SCR nucleotide sequences were obtained from NCBI using the accession numbers mentioned in Table 1. The length for RT-qPCR primers for all six genes were kept between 100–200 bp. Efficiency (E) of all these primers were calculated according to the equation: E = (10^[−1/sl^°^pe]^ −1) × 100 and relative standard curves for the transcripts were generated with serial dilutions of cDNA (1/5, 1/25, 1/125 and 1/625). Efficiencies for all the primers were found to be in the ideal range (90%-105%) for proper function of primer pairs for all six reference genes (Table 1). We also checked for the possibility of primer dimer or secondary structure formation using Gene Runner v. 3.05 software (Hastings Software, Inc.)^67^.

### RNA extraction and quantitative real-Time PCR (RT-qPCR)

Insects and insect tissues (i.e., eggs, larvae, and adults) were placed in 1.5 (eggs and neonates) or 2.0 ml tubes (other samples) containing one metal bead. The tubes were placed in metal blocks dipped in liquid nitrogen and appropriately balanced before being placed in SPEX SamplePrep 2010 Geno/Grinder^®^ machine (Metuchen, NJ). The machine was then switched on for 30 seconds at 1400 strokes min^−1^ to grind insect tissue materials into a fine powder for RNA extraction. Different SCR tissue samples (50–100 mg) were used for total RNA extraction using Qiagen RNeasy Mini Kit (Qiagen, Valencia, CA), following the manufacturer’s protocol and stored at −80 °C until use. The quantity of extracted RNA was estimated on a NanoDrop1000 (Thermo-Fisher Scientific, Wilmington, DE) and the quality was evaluated using 1% denaturing agarose gel electrophoresis. cDNAs were synthesized from 1 µg of total SCR RNA using the high capacity cDNA Reverse Transcriptase Kit (Applied Biosystems Inc., Foster City, CA). cDNA samples were diluted (1:50) before being used in RT-qPCR. Three independent biological replicates, each from three different insect cohorts were included in each RT-qPCR run. RT-qPCR was performed with SYBR Green Master Mix (iTaq™ Universal SYBR^®^ Green Supermix) on a Real-Time PCR System (Applied Biosystems Inc., Foster City, CA). The master mix (30 µl) contained 15 µl SYBR green, 9 µl water, 3 µl diluted (1:10) HKG primers (F + R) and 3 µl diluted (1:50) cDNA and was replicated in three wells of a 96-well plates using 10 μl/well. RT-qPCR was run at 95 °C for 30 seconds (Holding stage), at 95 °C for 15 seconds, then 60 °C for 1 minute (Cycling stage); followed by 95 °C for 15 seconds, 60 °C for 1 minute, and 95 °C for 15 seconds (Melt curve). The comparative 2^−ΔΔCt^ method^68^, which is calculated based on the difference between Ct values, was used to calculate the relative expression level of the six SCR HKGs in the samples as compared to control.

### Data analyses

Four different statistical algorithmic models were currently used for HKGs evaluation^17^: geNorm^16^, NormFinder^69^, BestKeeper^70^ and delta-Ct^71^. GeNorm assesses the expression stability value (M) for each gene and identifies the best pair of reference genes. This program is based on the mean pairwise variation between genes across all samples and the gene with the lowest M value is considered the most stable^16^. NormFinder estimates the standard deviation for each gene and compares it with the expression of the other genes. The gene with the lowest variation between intra- and inter-group comparisons is then considered the most stable^69^. BestKeeper is a data processing method based on crossing points that compares all genes across all samples and generates a stability index for each HKG^70^. The comparative delta-Ct method compares Ct values and the relative expression of ‘gene pairs’ with each sample^68,71^.

The mean Ct values of each sample generated in RT-qPCR for each HKG in each experiment were used as input data and analyzed using the web-based tool RefFinder (http://leonxie.esy.es/RefFinder/)^72^. The website integrates all four software algorithms, GeNorm^16^, NormFinder^69^, BestKeeper^70^, and the comparative delta-Ct method^71^ which provides a comprehensive stability index that ranks each HKG^72^. The lower the rank value, the more stable the HKG is. Pairwise variation (V), determined by geNorm, was used to determine the optimal number of reference genes for accurate RT-qPCR normalization. Vn/Vn + 1 indicated the pairwise variation between two sequential normalization factors, and a cutoff threshold of Vn/Vn + 1 = 0.15 was used for valid normalization^16^.

## Supplementary information

Dataset 1
