# Supplementary material for: Evaluation of reference genes for real-time quantitative PCR analysis in southern corn rootworm, Diabrotica undecimpunctata howardi (Barber)
Source: Sci Rep. 2019 Jul 24;9:10703. doi: 10.1038/s41598-019-47020-y (PMC6656754; doi:10.1038/s41598-019-47020-y)
Supplement: Supplementary file 1 — Dataset 1 [file 41598_2019_47020_MOESM1_ESM.pdf]

Evaluation of reference genes for real-time quantitative PCR analysis in southern corn rootworm, *Diabrotica undecimpunctata howardi* (Barber)

Saumik Basu [a], Adriano E. Pereira [a, *], Daniele H. Pinheiro [c], Haichuan Wang [d], Arnubio Valencia-Jiménez [e], Blair D. Siegfried [f], Joe Louis [a, b], Xuguo 'Joe' Zhou [g], and Ana Maria Vélez [a]

(Saumik Basu and Adriano E. Pereira contributed equally to the manuscript)

[a] Department of Entomology, University of Nebraska-Lincoln, Lincoln, NE 68583

[b] Department of Biochemistry, University of Nebraska-Lincoln, Lincoln, NE 68583

[c] Federal University of Lavras, Lavras, MG, Brazil

[d] Department of Agronomy and Horticulture, University of Nebraska-Lincoln, Lincoln, NE, 68583-0915

Supporting information

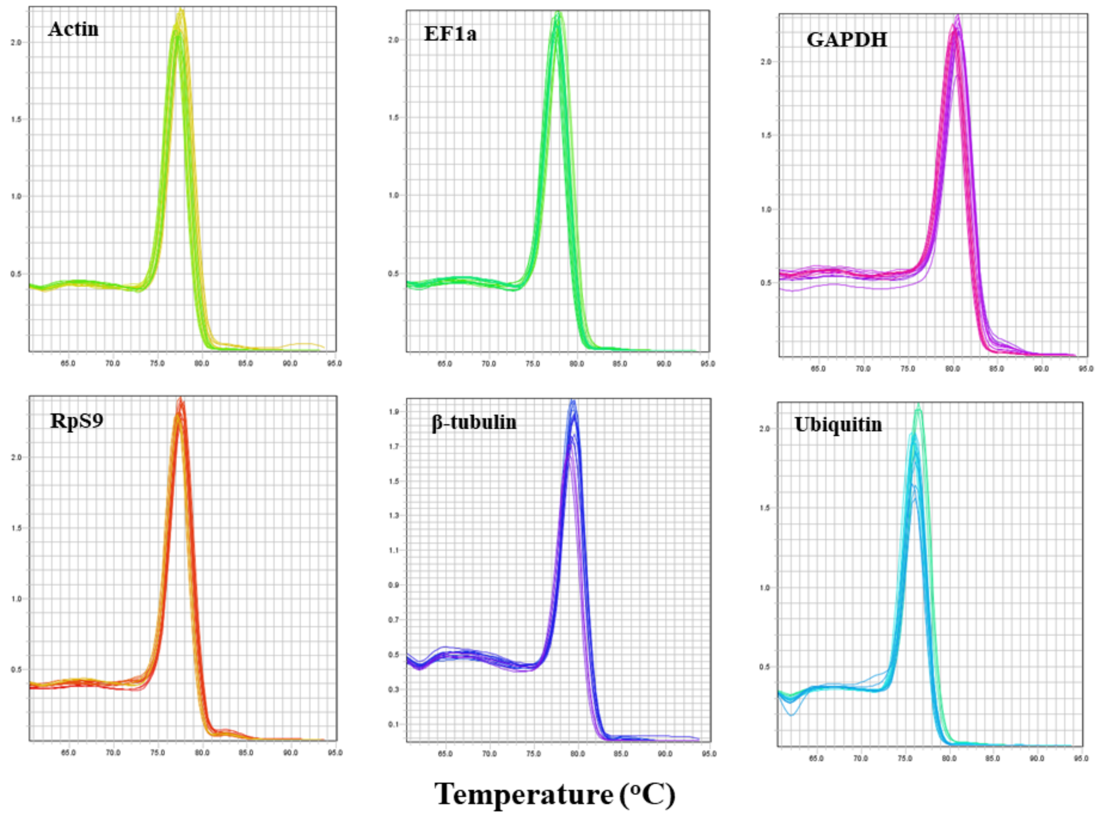

Supplemental Figure 1. Melting curve analyses showing the presence of single peak for the six genes evaluated (*Actin*, *EF1α*, *GAPDH*, *RpS9*, *Tubulin* and *Ubi*) in SCR which confirmed RT-qPCR amplification by their specific primers.

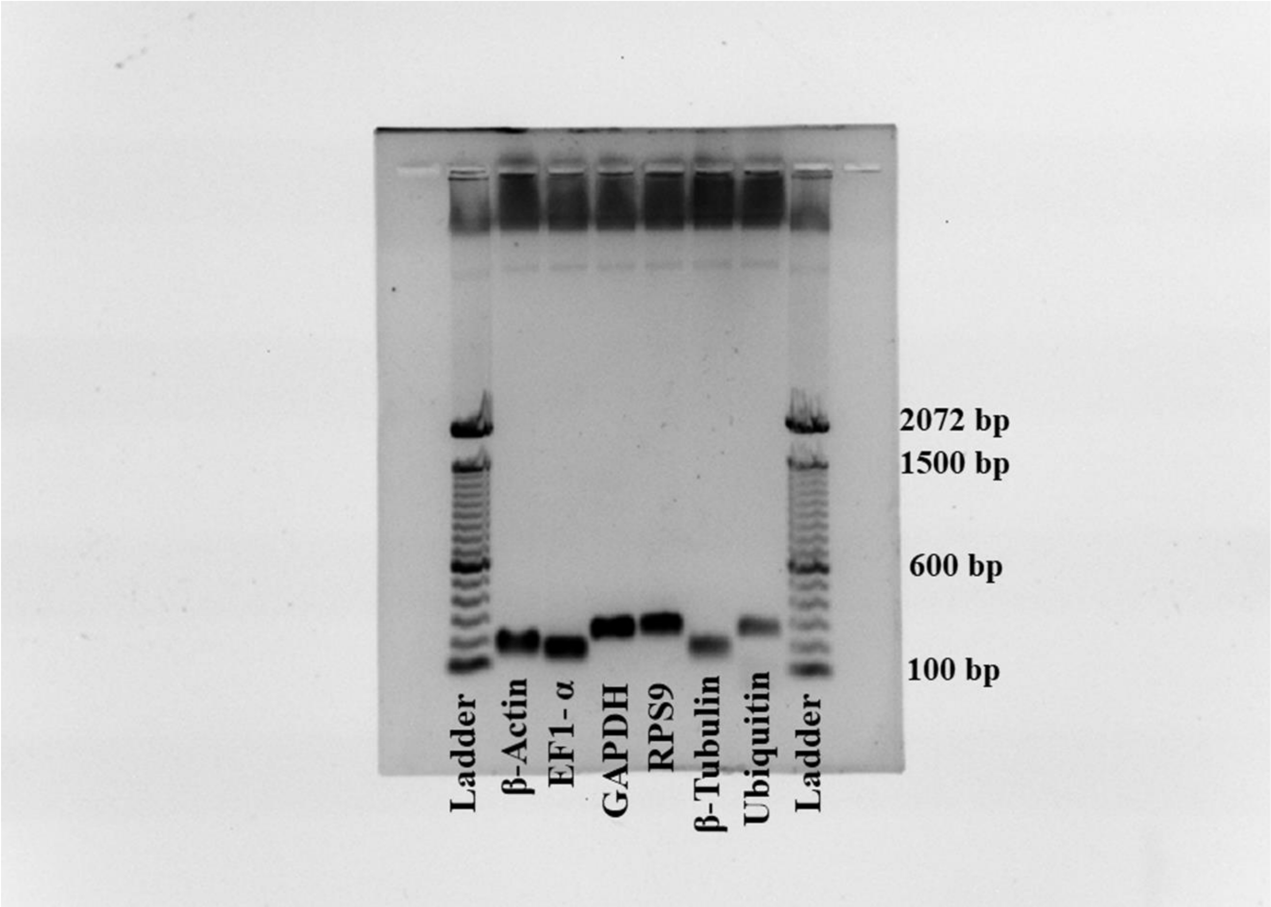

Supplemental Figure 2. PCR amplification of six SCR HKGs, *Actin*, *EF1α*, *GAPDH*, *RpS9*, *Tubulin* and *Ubi* using gene-specific primers.

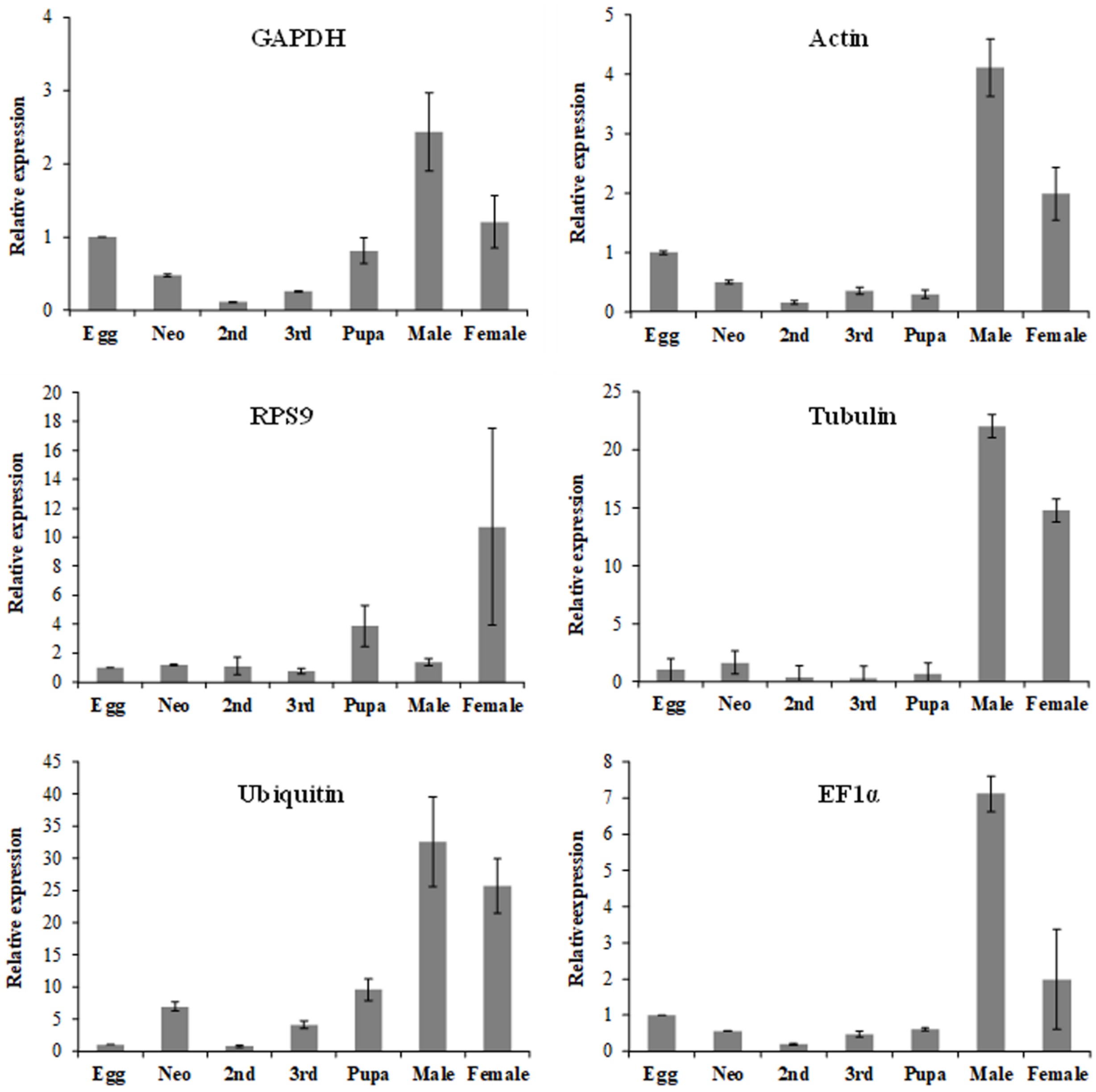

Supplemental Figure 3. Relative gene expression of SCR HKGs *Actin*, *EF1α*, *GAPDH*, *RpS9*, *Tubulin* and *Ubi* performed in the different life stages egg, neonate, second instar, third instar, pupa, adult male and female, using RT-qPCR.
